# The Proteasome Inhibitor Bortezomib Controls Indoleamine 2,3-Dioxygenase 1 Breakdown and Restores Immune Regulation in Autoimmune Diabetes

**DOI:** 10.3389/fimmu.2017.00428

**Published:** 2017-04-13

**Authors:** Giada Mondanelli, Elisa Albini, Maria T. Pallotta, Claudia Volpi, Lucienne Chatenoud, Chantal Kuhn, Francesca Fallarino, Davide Matino, Maria L. Belladonna, Roberta Bianchi, Carmine Vacca, Silvio Bicciato, Louis Boon, Giovanni Ricci, Ursula Grohmann, Paolo Puccetti, Ciriana Orabona

**Affiliations:** ^1^Section of Pharmacology, Department of Experimental Medicine, University of Perugia, Perugia, Italy; ^2^INSERM U1013, Hôpital Necker-Enfants Malades, Université Paris Descartes, Paris, France; ^3^VL37 Inc., Cambridge, MA, USA; ^4^Department of Life Sciences, University of Modena and Reggio Emilia, Modena, Italy; ^5^Bioceros BV, Utrecht, Netherlands; ^6^Animal Facility of the University of Perugia, Perugia, Italy

**Keywords:** tryptophan metabolism, indoleamine 2,3-dioxygenase 1 enzyme, dendritic cells, proteasome, autoimmune diabetes, immune regulation, anti-CD3 antibody

## Abstract

Bortezomib (BTZ) is a first-in-class proteasome inhibitor approved for the therapy of multiple myeloma that also displays unique regulatory activities on immune cells. The enzyme indoleamine 2,3-dioxygenase 1 (IDO1) is a tryptophan metabolizing enzyme exerting potent immunoregulatory effects when expressed in dendritic cells (DCs), the most potent antigen-presenting cells capable of promoting either immunity or tolerance. We previously demonstrated that, in inflammatory conditions, IDO1 is subjected to proteasomal degradation in DCs, turning these cells from immunoregulatory to immunostimulatory. In non-obese diabetic (NOD) mice, an experimental model of autoimmune diabetes, we also identified an IDO1 defect such that the DCs do not develop tolerance toward pancreatic islet autoantigens. We found that BTZ rescues IDO1 protein expression *in vitro* in a particular subset of DCs, i.e., plasmacytoid DCs (pDCs) from NOD mice. When administered *in vivo* to prediabetic mice, the drug prevented diabetes onset through IDO1- and pDC-dependent mechanisms. Although the drug showed no therapeutic activity when administered alone to overtly diabetic mice, its combination with otherwise suboptimal dosages of autoimmune-preventive anti-CD3 antibody resulted in disease reversal in 70% diabetic mice, a therapeutic effect similar to that afforded by full-dosage anti-CD3. Thus, our data indicate a potential for BTZ in the immunotherapy of autoimmune diabetes and further underline the importance of IDO1-mediated immune regulation in such disease.

## Introduction

Dendritic cells (DCs) have an important role as islet antigen-presenting cells in the initiation of autoimmune diabetes in the non-obese diabetic (NOD) mouse, in which defective tryptophan catabolism by immune regulatory indoleamine 2,3-dioxygenase 1 (IDO1) contributes to an overall impaired tolerogenesis. Functional flexibility represents a feature of plasmacytoid DCs (pDCs), capable of inducing either immune activation or tolerance, depending on environmental conditions (1–5). Protein expression of IDO1—and thus the functional balance between immunity and tolerance—can greatly vary in pDCs in response to transcriptional *Ido1* inducers as well as post-transcriptional mechanisms. Splenic pDCs from conventional (e.g., C57BL/6 or BALB/c) mice express basal yet low levels of IDO1 protein, which can be increased by interferon (IFN)-γ, the standard IDO1 transcriptional inducer. As a result, unstimulated splenic pDCs are immunogenic, whereas IFN-γ-stimulated pDCs are tolerogenic (6, 7). The post-transcriptional mechanisms include, instead, proteasomal degradation of the enzyme, which is driven by inflammatory IL-6 and results in sustained inflammation and impaired tolerogenesis (4, 6–8).

In NOD mice, IDO1 expression and activity in pDCs are almost negligible in response to IFN-γ (9, 10), due to an impaired IFN-γ signaling activity (9) and abnormal regulatory proteolysis of the enzyme sustained by high levels of IL-6 (10). In agreement with this, systemic administration of IL-6-neutralizing antibodies results in marked suppression of the diabetic condition in NOD animals (11). Moreover, inflammatory cytokines, such as IFN-γ and tumor necrosis factor-α, induce the synthesis of catalytic subunits of the immunoproteasome—LMP2/β1i, MECL1/β2i, and LMP7/β5i—which replace their constitutive proteasome counterparts in hematopoietic cells (12). Selective inhibition of the β5i subunit has been shown to control disease progression in a series of experimental autoimmune disorders and promote long-term allograft survival (13–17).

Bortezomib (BTZ) is a reversible proteasome inhibitor that possesses immune regulatory activities that span various cellular processes of T and DCs essential for the development of adaptive immune responses (18). BTZ suppresses T-cell activation and production of cytokines, inhibits T-cell mobilization, suppresses T-cell growth and proliferation, and induces T-cell apoptosis. In DCs, BTZ suppresses maturation and function, cytokine production, and induces apoptosis. The drug can also inhibit nuclear translocation of NF-κB family members in DCs, thus supporting a pleiotropic function of BTZ in regulating DC function at the interface between immunity and tolerance (19).

Here, we report that BTZ confers tolerogenic effects on pDCs from NOD mice, resulting in enhanced generation of regulatory T cells and preventing diabetes onset in mice through an IDO1-dependent mechanism. A combination of BTZ and otherwise suboptimal dosages of anti-CD3 was found to rescue normoglycemia in overtly diabetic NOD mice.

## Animals and Methods

### Animals

Female BALB/c and NOD/MrkTac female mice, aged 6–16 weeks, were purchased from Charles River Breeding Laboratories (Calco, Milan, Italy) and Taconic (Albany, NY, USA), respectively. IDO1-deficient (*Ido1^−/−^*) NOD female mice were bred at the animal facility of the University of Perugia.

### Cell Purification and *In Vitro* Treatments

Cells were isolated from pancreatic lymph nodes (pLNs) and pancreata by digestion with collagenase type IV (Sigma-Aldrich, St. Louis, MO, USA); total peritoneal cells (PeCs) were isolated from the peritoneal washes by centrifugation. Unfractionated cell populations were promptly used for cytofluorimetric analysis or incubated at 37°C for 16 h for cytokines and/or kynurenines detection in culture supernatants. All purification procedures involving pDCs and conventional DCs (cDCs, CD11c^+^ CD8^−^) have previously been described (6, 7, 9). The purity of DC populations was superior to 90%. Purified pDCs were exposed at 37°C to 200 U/ml of recombinant IFN-γ (R&D Systems, Minneapolis, MN, USA) or to 10 nM of BTZ (LC Laboratories, MA, USA) in the presence or absence of 4 μM 1-methyl-DL-tryptophan (1-MT; Sigma-Aldrich, St. Louis, MO, USA), a standard IDO1 inhibitor. In selected experiments, pDCs pretreated as described above, were co-cultured (2:1 cell ratio) with CD4^+^CD25^−^ T lymphocytes purified from pLNs, and maintained at 37°C for 96 h, before harvesting the supernatants for the cytokine analysis and the cells for cytofluorimetric analysis.

### Western Blot, ELISA, and Cytofluorimetric Analyses

Indoleamine 2,3-dioxygenase 1 protein expression was investigated in pDCs by immunoblot with a rabbit polyclonal anti-mouse IDO1 antibody raised in our laboratory (20). Anti-β-tubulin antibody (Sigma-Aldrich) was used as a normalizer. Mouse IL-6, IL-17A, IFN-γ, IL-10, and TGF-β1 were measured in culture supernatants by ELISA with specific kits (eBioscience, Inc., San Diego, CA, USA; Promega Italia, S.r.l., Milano, Italy). An ELISA–based TransAM Flexi NF-κB Family Kit (Active Motif, Rixensart, Belgium) was used to monitor activity of NF-κB family members, as described (6, 7, 10). Cytofluorimetric assays were conducted using the specific fluorochrome-labeled antibodies for murine CD19, CD5, CD1d, CD4, Foxp3, and RORγt (Biolegend, BD Pharmingen, eBiocience Inc., San Diego, CA, USA; Miltenyi Biotech, Germany), as described (6). For intracellular staining of Foxp3 and RORγt, cells were previously permeabilized by the Fix/Perm Buffer set (Biolegend, San Diego, CA, USA).

### Kynurenine Assay

The functional activity of IDO1 was measured *in vitro* in terms of the ability to metabolize tryptophan (Trp) to kynurenine (Kyn), whose concentration was measured by high-performance liquid chromatography (HPLC) in culture supernatants after the addition of 100 µM Trp (20). Systemic Kyn/Trp ratio was determined in deproteinated sera of mice by measuring Kyn and Trp concentrations by HPLC.

### *In Vivo* Treatment and Histopathology in NOD Mice

Bortezomib was administered i.p. at the dose of 0.10 or 0.25 mg/kg, a dose well below the drug’s maximum tolerated dose, i.e., 0.5 mg/kg (21), every other day for 2 weeks in prediabetic or in overtly diabetic NOD mice. In selected experiments, overtly diabetic mice were coadministered with BTZ and anti-CD3 (145-2C11) F(ab′)_2_ fragments (22) (provided by Lucienne Chatenoud, Université Paris Descartes, Paris) i.v. at a suboptimal dose of 10 µg/day, for five consecutive days. Selected groups of mice received the standard IDO1 inhibitor 1-MT in their drinking water at the concentration of 2 mg/ml. *In vivo* depletion of pDCs occurred 1 week after commencing BTZ treatment, by injecting prediabetic NOD mice with 250 μg/mouse of 120G8 (IgG2a isotype) antibody i.p., as described (23). Animals were either monitored for glycemia or sacrificed for *ex vivo* analyses at different times of drug treatment(s). For histopathology, 3–4 µm of paraffin-embedded sections of pancreata (5/organ) were stained with hematoxylin and eosin and analyzed by light microscopy. Insulitis scoring was done according to standard criteria as described (24).

### Skin Test Assay

A skin test assay was used for measuring delayed-type hypersensitivity (DTH) in response to challenge in the footpad with the diabetogenic H-2K^d^-restricted IGRP peptide (VYLKTNVFL), as described (7), using prediabetic NOD mice as recipients. For *in vivo* immunization, 3 × 10^5^ peptide-loaded NOD cDCs, combined with a minority fraction (5%) of peptide-loaded NOD pDCs, and pre-incubated *in vitro* as indicated in the relevant figure legends, were injected subcutaneously into recipient mice. Two weeks later, a DTH response was measured to intrafootpad challenge with the eliciting peptide, and results were expressed as the change in footpad weight of peptide-injected footpads over that of vehicle-injected (internal control) counterparts (4, 6, 9).

### Statistical Analysis

*In vitro* studies comparing more than two experimental conditions were analyzed by ANOVA test followed by Dunnett’s or Bonferroni test, while Student’s *t*-test was used for the analysis of *in vitro* results comparing two conditions. In the *in vivo* skin test assay, a paired Student’s *t*-test was used for statistical analysis by comparing the weight change of the experimental footpads with vehicle-injected counterpart (9). Diabetes incidence was plotted by using the Kaplan–Meier method, i.e., a non-parametric cumulative survival plot. The statistical comparison between the curves was performed by using the log-rank (Mantel–Cox) test.

## Results

### BTZ Confers IDO1-Dependent Tolerogenic Effects on pDCs from NOD Mice

**Figure 1:**
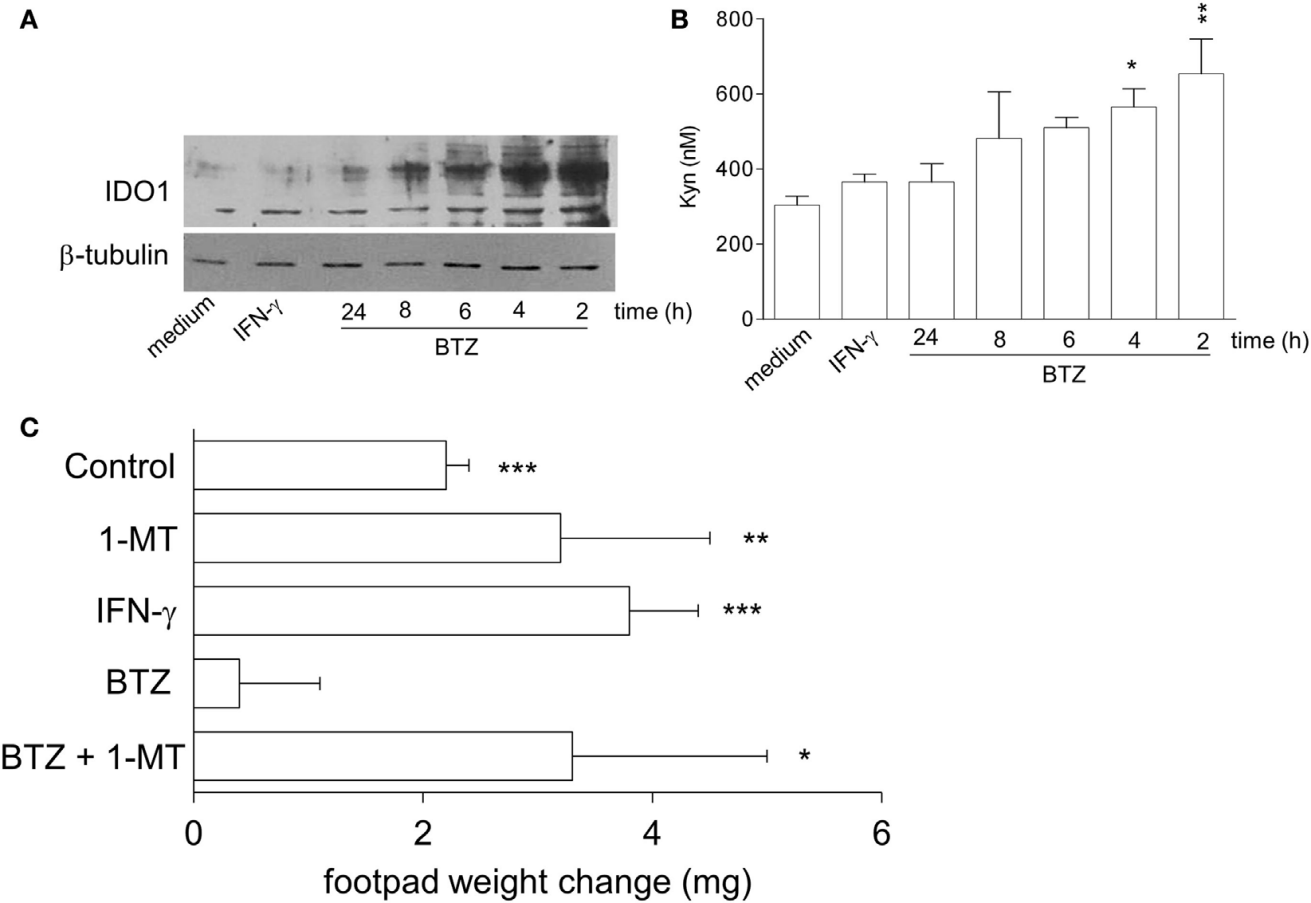
**Bortezomib (BTZ) restores the tolerogenic potential of prediabetic non-obese diabetic (NOD) plasmacytoid DCs (pDCs) in an indoleamine 2,3-dioxygenase 1 (IDO1)-dependent fashion**. **(A)** IDO1 immunoblot analysis of cell lysates from splenic pDCs purified from prediabetic NOD mice incubated with BTZ for the indicated time, interferon (IFN)-γ or medium alone for 24 h. One representative experiment out of three is shown. **(B)** IDO1 activity measured in terms of kynurenine production in cell supernatant of pDCs incubated as in panel **(A)**. *p < 0.05; **p < 0.01 (one-way ANOVA and Dunnett’s multiple comparison test). Data are presented as means ± SD from triplicate samples. **(C)** IGRP-pulsed prediabetic NOD conventional DCs in combination with a minority fraction (5%) of NOD pDCs with no conditioning (control) or *in vitro* conditioned with 1-MT, IFN-γ, BTZ alone, or in combination with 1-MT, were transferred into NOD recipient mice (*n* = 6/group) to be assayed for skin reactivity to the eliciting peptide. Analysis of skin test reactivity is presented as change in footpad weight. *p < 0.05; **p < 0.01; ***p < 0.001 (paired Student’s *t*-test; weight change of experimental vs. control footpad).

We initially examined whether proteasome inhibition by BTZ would upregulate IDO1 expression and function in pDCs from NOD mice. *In vitro*, splenic pDCs, purified from prediabetic NOD mice, were incubated with 10 nM BTZ or 200 U/ml IFN-γ and, at different times, cell lysates and supernatants were analyzed for IDO1 protein and Kyn production, respectively. As expected, IFN-γ failed to upregulate IDO1 protein (Figure 1A) and activity (Figure 1B) in NOD pDCs. By contrast, pDCs conditioned with BTZ showed higher IDO1 expression (Figure 1A) and produced significantly higher levels of Kyn (Figure 1B). Although the increase in IDO1 protein expression was rather short-lived, treatment of NOD pDCs with BTZ induced the appearance of high molecular-weight IDO1 proteins (Figure 1A), corresponding to poliubiquitinated forms of the protein. We also found significantly higher expression of *Socs3—*the IL-6-induced molecular driver of IDO1 proteasomal degradation—and of the immunoproteasome subunits in NOD, compared to BALB/c pancreata (Figure S1A in Supplementary Material) and in prediabetic vs. diabetic pDCs, purified from NOD pLNs (Figure S1B in Supplementary Material). Interestingly, prediabetic NOD pDCs were also featured by a higher expression of the gene set involved in the pro-inflammatory NF-κB pathway (Figure S1C in Supplementary Material), suggesting a more inflammatory-prone phenotype of pDCs in the early phase of the disease.

*In vivo*, NOD female mice were sensitized with the diabetogenic IGRP peptide loaded on highly immunogenic NOD cDCs. The cDCs were administered alone or in combination with a minority fraction of NOD pDCs, treated with control media, IFN-γ, or BTZ (the latter alone or in combination with the IDO1 inhibitor 1-MT). Two weeks later, *in vivo* IGRP-specific immune reactivity was assayed. As expected, the priming ability of immunostimulatory cDCs was not affected by the presence of untreated or IFN-γ-stimulated NOD pDCs. However, co-administration of BTZ-treated pDCs ablated the IGRP-specific reactivity elicited by cDCs. The suppressive effect of BTZ-conditioned pDCs was, however, lost when 1-MT treatment was applied only in concurrence with BTZ exposure (Figure 1C). These data clearly show that BTZ—unlike IFN-γ—regulates the quantitative and qualitative expression of IDO1 protein in prediabetic NOD pDCs, restoring their tolerogenic potential *in vivo*. In addition to the signaling defect previously described (9), post-transcriptional anomalies were found to contribute to the failure of IFN-γ to enhance IDO1 expression in NOD mice.

### BTZ-Conditioned NOD pDCs Favor the Differentiation of Regulatory Rather Than Effector T Cells *In Vitro*

**Figure 2:**
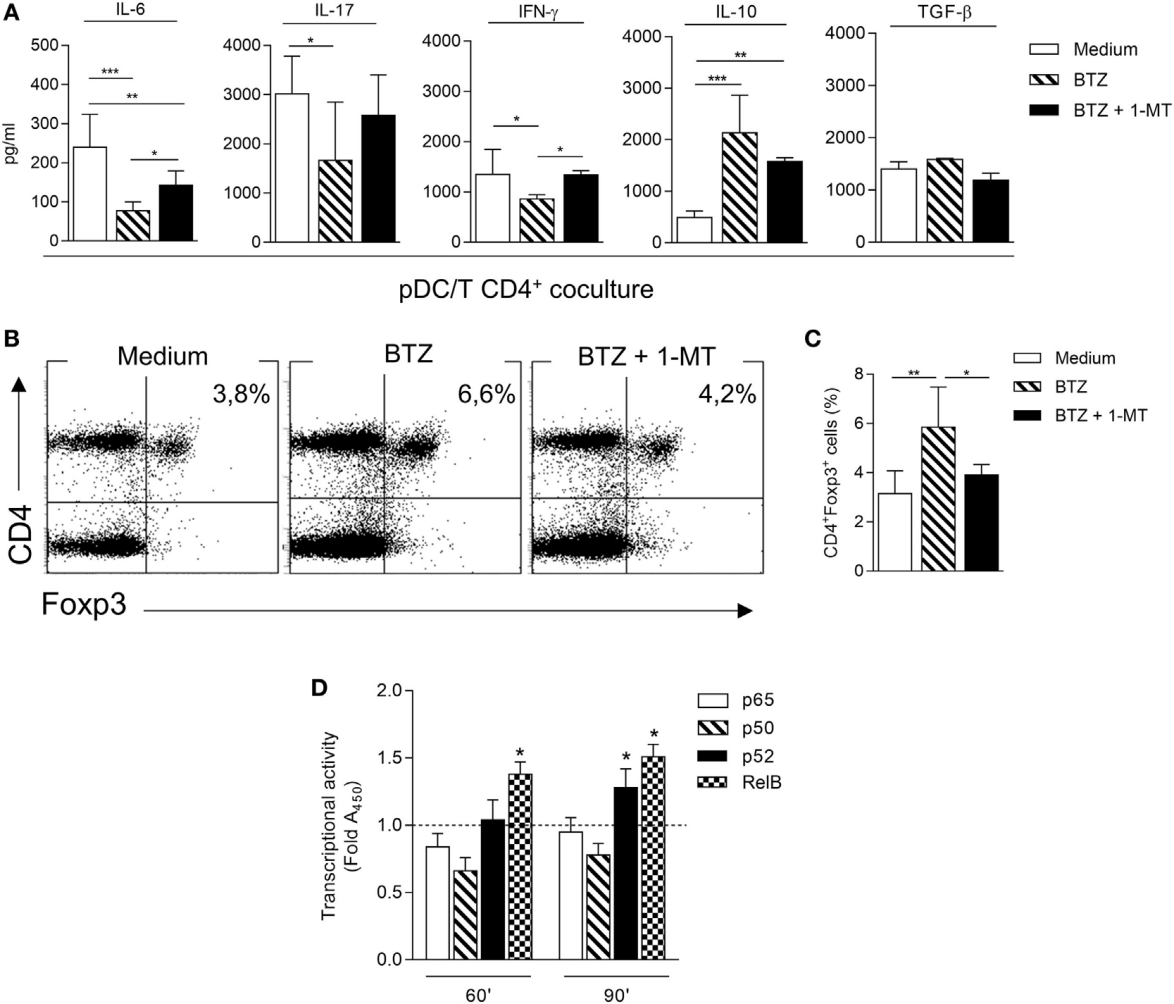
**Bortezomib (BTZ) conditions inflammatory-prone non-obese diabetic plasmacytoid DCs (pDCs) toward immunoregulatory signals**. **(A)** Secretion of IL-6, IL-17, interferon (IFN)-γ, IL-10, and TGF-β in supernatant of pDC-CD4^+^ T cell cocultures, where pDCs were pretreated with BTZ alone or in combination with 1-MT, or left untreated (medium) for 24 h. *p < 0.05; **p < 0.01; ***p < 0.001 (one-way ANOVA and Dunnett’s multiple comparison test). Results in panel **(A)** are representative of three different experiments (mean ± SD). **(B)** Representative flow cytometric dot plots illustrating the frequency of CD4^+^Foxp3^+^ cells among CD4^+^CD25^−^ T cells cultured for 4 days together with pDCs treated as in panel **(A)**. **(C)** Percentage of CD4^+^Foxp3^+^ cells in a CD4^+^CD25^−^ T cells population cultured for 4 days together with pDCs treated as in panel **(A)**. *p < 0.05; **p < 0.01 (one-way ANOVA followed by Bonferroni multiple comparison test). Results are representative of three different experiments (mean ± SD). **(D)** ELISA detection of p65, p50, p52, and RelB subunits of NF-κB in nuclear extracts of pDCs untreated (dotted line) or treated with BTZ for 60 and 90 min. Results are presented as fold change of the absorbance at 450 nm (Fold A_450_). *p < 0.05 (paired Student’s *t*-test; treated vs. untreated samples). Data are presented as means ± SD from triplicate samples.

Next, we investigated the ability of BTZ-conditioned NOD pDCs to affect T-cell differentiation and/or function when cocultured with CD4^+^ T cells. In the pDC-CD4^+^ T-cell cocultures, BTZ-pretreated pDCs significantly decreased IL-6, IFN-γ (as T helper 1 marker), and IL-17 (as T helper 17 marker) in the coculture supernatant, and IL-10 but not TGF-β was instead increased (Figure 2A). An analogous cytokine profile was observed after BTZ conditioning of the pDCs alone (Figure S2 in Supplementary Material). Interestingly, the co-presence of 1-MT at the time of pDCs exposure to BTZ selectively negated the drug effect on the pro-inflammatory cytokine (i.e., IL-6 and IFN-γ) secretion profile (Figure 2A), suggesting that IDO1 upregulation by BTZ could play a role in controlling those cytokines. Cytofluorimetric analysis conducted at the end of the coculture showed that BTZ-pretreated pDCs induced a twofold increase in the percentage of CD4^+^Foxp3^+^ T cells, an effect negated by 1–MT, the IDO1 inhibitor (Figures 2B,C).

In immune cells, BTZ has been shown to downregulate the production of several pro-inflammatory cytokines, presumably *via* inhibition of NF-κB activation (25, 26). Moreover, prediabetic pDCs showed a higher expression of the pro-inflammatory NF-κB pathway compared to diabetic pDCs (Figure S1C in Supplementary Material). To evaluate any role for impaired NF-κB activation by BTZ in our setting, pDCs purified from NOD mice were incubated with 10 nM BTZ or medium alone and the activation of NF-κB was evaluated in nuclear cell lysates after 60 and 90 min. A significant reduced nuclear translocation of p50 and p65 canonical subunits could be observed after 60 min in BTZ-treated as compared to untreated NOD pDCs; at a later time (90 min) a significant nuclear translocation of the non-canonical subunits p52 and RelB could be observed (Figure 2D). The presence of BTZ in the cell culture significantly modified the transcriptional activity of NF-κB, shifting the balance toward the non-canonical pathway, which we have previously shown as being involved in the transcription of *Ido1* (6, 7).

Overall, these data suggested that the BTZ exposure of inflammation-prone pDCs—such as those from NOD mice (27, 28)—would tip the balance between inflammatory and immunoregulatory signals in favor of the latter, *via* upregulation of the IDO1 mechanism and also *via* correction of a dysfunctional NF-κB activity.

### BTZ Prevents Diabetes Development in NOD Mice

**Figure 3:**
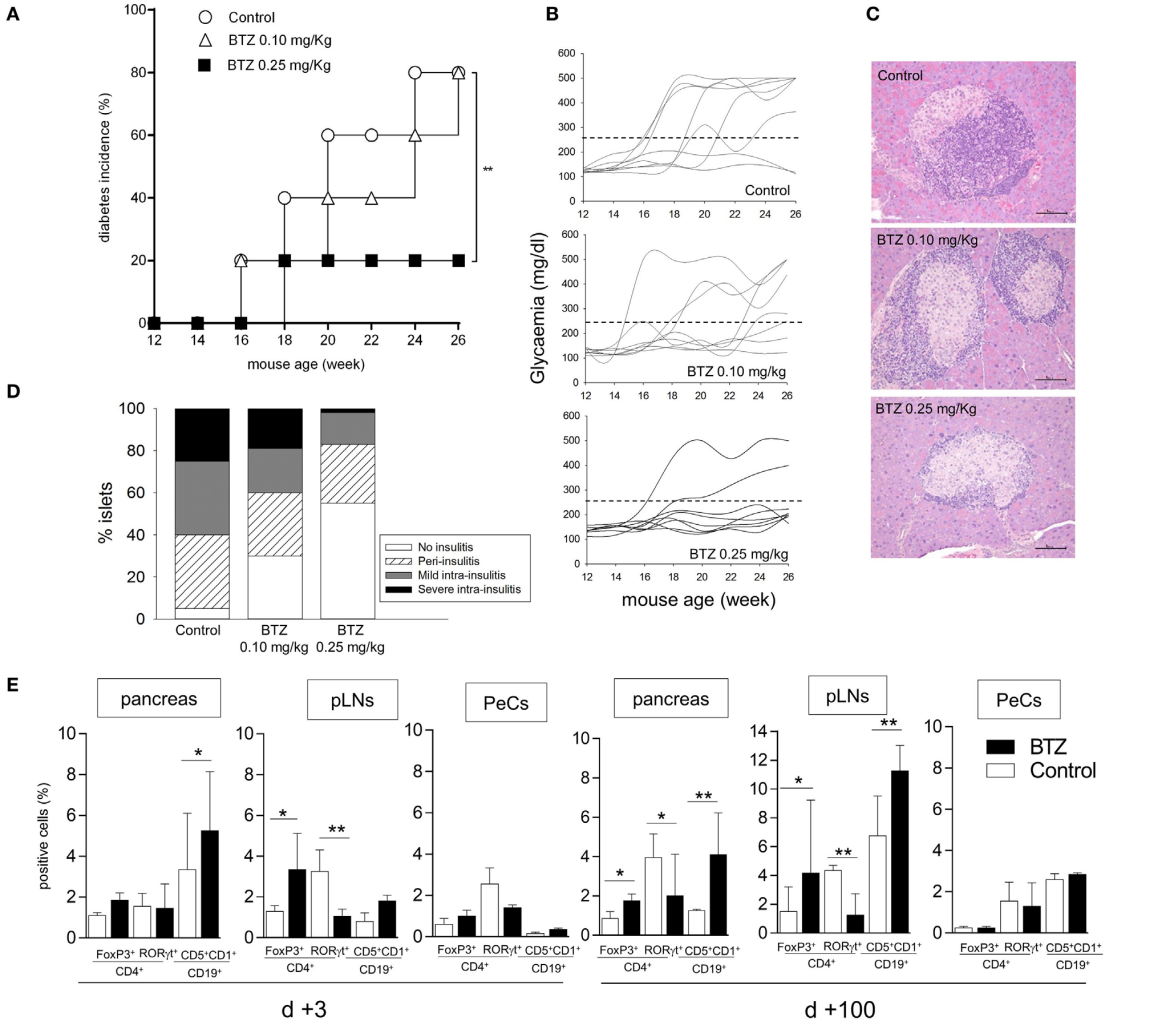

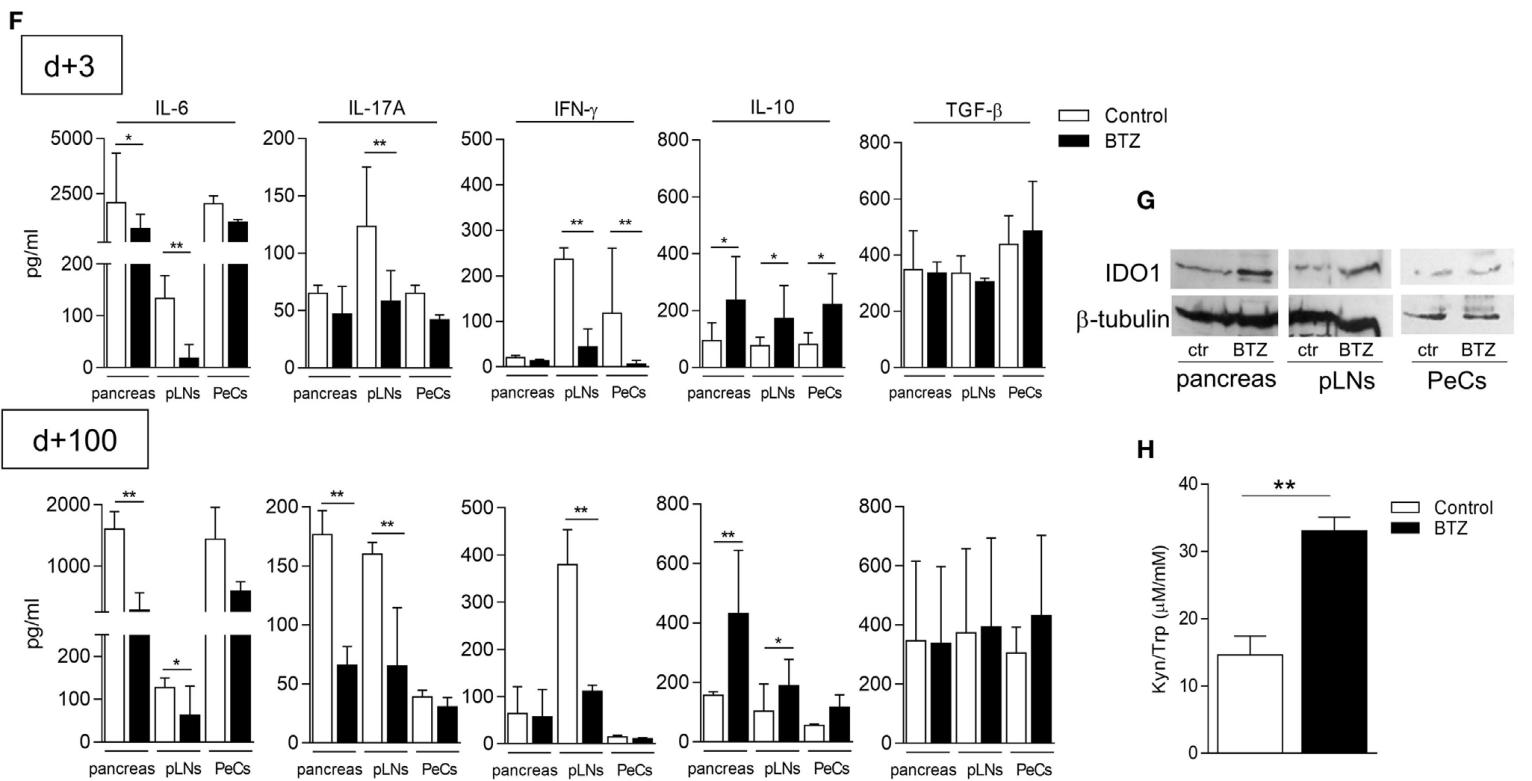
**Administration of bortezomib (BTZ) prevents the development of autoimmune diabetes in non-obese diabetic (NOD) mice**. **(A)** Prediabetic female NOD mice (*n* = 8) were intraperitoneally administered with vehicle alone (control; white circles), BTZ at 0.10 mg/kg (white triangles) or 0.25 mg/kg (black squares), and blood glucose was monitored over time. Diabetes was diagnosed in mice with blood glucose level ≥250 mg/dl and results are reported as diabetes incidence. **p < 0.01 (log-rank Mantel–Cox test). **(B)** Single blood glucose concentrations of NOD mice treated as in panel **(A)**. **(C)** Pancreatic histology of mice treated as in panel **(A)** (scale bar, 100 µm; representative islet area for each group are shown). **(D)** Islets were scored to record percentages of islets of a given score over total number of islets (20–30/pancreas). One experiment is depicted of three with similar results. **(E)** Cytofluorimetric analysis of the frequency of Foxp3^+^ and RORγt^+^ cells among CD4^+^ T cell population and CD19^+^CD5^+^CD1d^+^ cells in pancreas, pancreatic lymph nodes (pLNs) and peritoneal cells (PeCs) from prediabetic female NOD mice treated with BTZ at 0.25 mg/kg or vehicle alone (control) and sacrificed at day +3 or day +100 after the last drug treatment. *p < 0.05 and **p < 0.01 (unpaired Student’s *t*-test, BTZ-treated vs. control mice). **(F)** Detection of IL-6, IL-17A, IFN-γ, IL-10, and TGF-β in supernatant of unfractionated cells from pancreas, pLNs, and PeCs of mice treated as in panel **(E)** and cultured for 24 h. *p < 0.05 and **p < 0.01 (unpaired Student’s *t*-test, BTZ-treated vs. control mice for each cell type). **(G)** Indoleamine 2,3-dioxygenase 1 immunoblot analysis of cell lysates from pancreas, pLNs, and PeCs of mice treated as in panel **(E)**. One representative experiment out of three is shown. **(H)** Kynurenine to tryptophan ratio (Kyn/Trp) measured in sera from mice treated as in panel **(E)**. **p < 0.01 (unpaired Student’s *t*-test, BTZ-treated vs. control mice). Data are presented as means ± SD for each group (*n* = 8 per group).

Because IDO1 has been shown to play a major role in the protection from the experimental autoimmune diabetes (10, 24, 29, 30), we performed *in vivo* experiments to ascertain whether BTZ could exert protective effects in NOD mice. Groups of female NOD mice in prediabetes were thus administered BTZ i.p. at 0.10 or 0.25 mg/kg every other day for 2 weeks, a well-tolerated regimen, and glycemia was regularly measured. Over time, approximately 80% of control mice, and mice given the lower BTZ dose, developed overt diabetes, with glycemia values exceeding 250 mg/dl. By contrast, only 20% of mice administered BTZ at 0.25 mg/kg became hyperglycemic (Figure 3A), and glycemia values of protected mice remained in the range of 120–170 mg/dl over time (Figure 3B). In mice administered 0.25 mg/kg BTZ, histology of the pancreata revealed the presence of preserved islets, without invasive insulitis or with minimum or no peri-insulitis. The great majority of islets of the control and mice receiving the lower BTZ dose was characterized by immune cell infiltration (Figures 3C,D).

In order to investigate the *in vivo* effects of BTZ on immune cells, additional groups of mice, receiving 0.25 mg/kg BTZ or vehicle treatment, were sacrificed at different times (day +3 and day +100 after the last drug administration) and used as source of pancreas, pLNs, and PeCs—peritoneum being the site of BTZ injection—to be analyzed for T and B cell populations, cytokine production, and IDO1 expression. Cytofluorimetric analyses showed that, at 3 and more so 100 days, the drug significantly increased percentages of CD4^+^ Foxp3**^+^** (Treg) and CD19^+^ CD5^+^ CD1d^+^ (Breg) cells in pancreas and pLNs but not in PeCs. In parallel, a decrease in CD4^+^ RORγt^+^ T cells (i.e., Th17 subset) was found in pLNs and later on (day +100) in pancreata (Figure 3E). Cytokine analysis of the same sources cultured for 24 h revealed most evident changes in IL-6 production, which started to decrease as soon as on day +3 in the pancreas of BTZ-treated mice relative to controls and in pLNs as well, though later in time (day +100; Figure 3F). An early (day +3) IL–17A decrease was also found in cultured pLNs and it persisted longer (day +100), whereas, in the pancreas, IL-17A decreased only later in time (day +100). IFN-γ decreased early in pLNs and PeCs and the effect persisted in pLNs up to day +100. Release of IL-10, but not of TGF-β, was increased on day +3 in all supernatants from whichever source and it remained increased in the pancreas and pLNs on day +100 (Figure 3F). IDO1 protein expression appeared to be upregulated in pancreas and pLNs, but not in PeCs from mice administered BTZ (Figure 3G). Moreover, the Kyn/Trp ratio increased in sera from mice treated with BTZ (Figure 3H).

Thus, our *in vivo* data indicated that proteasomal inhibition is effective in preventing development of autoimmune diabetes in a significant percentage of mice and that the protective effect is accompanied by a decrease in pro-inflammatory effector (Th17) cells and cytokines (IL-6, IL-17A, and IFN-γ), as well as by an increase in regulatory (Treg and Breg) cells and anti-inflammatory IL-10. Moreover, the *in vivo* treatment with BTZ upregulated IDO1 protein in the pancreas and pLNs and increased the systemic Kyn/Trp ratio. Importantly, changes were most evident in the pancreas, in immune cells draining (pLNs), and in proximity of the pancreas (namely, peritoneum, which was also the injection site).

### Prevention of Diabetes by BTZ Requires IDO1 and pDCs

**Figure 4:**
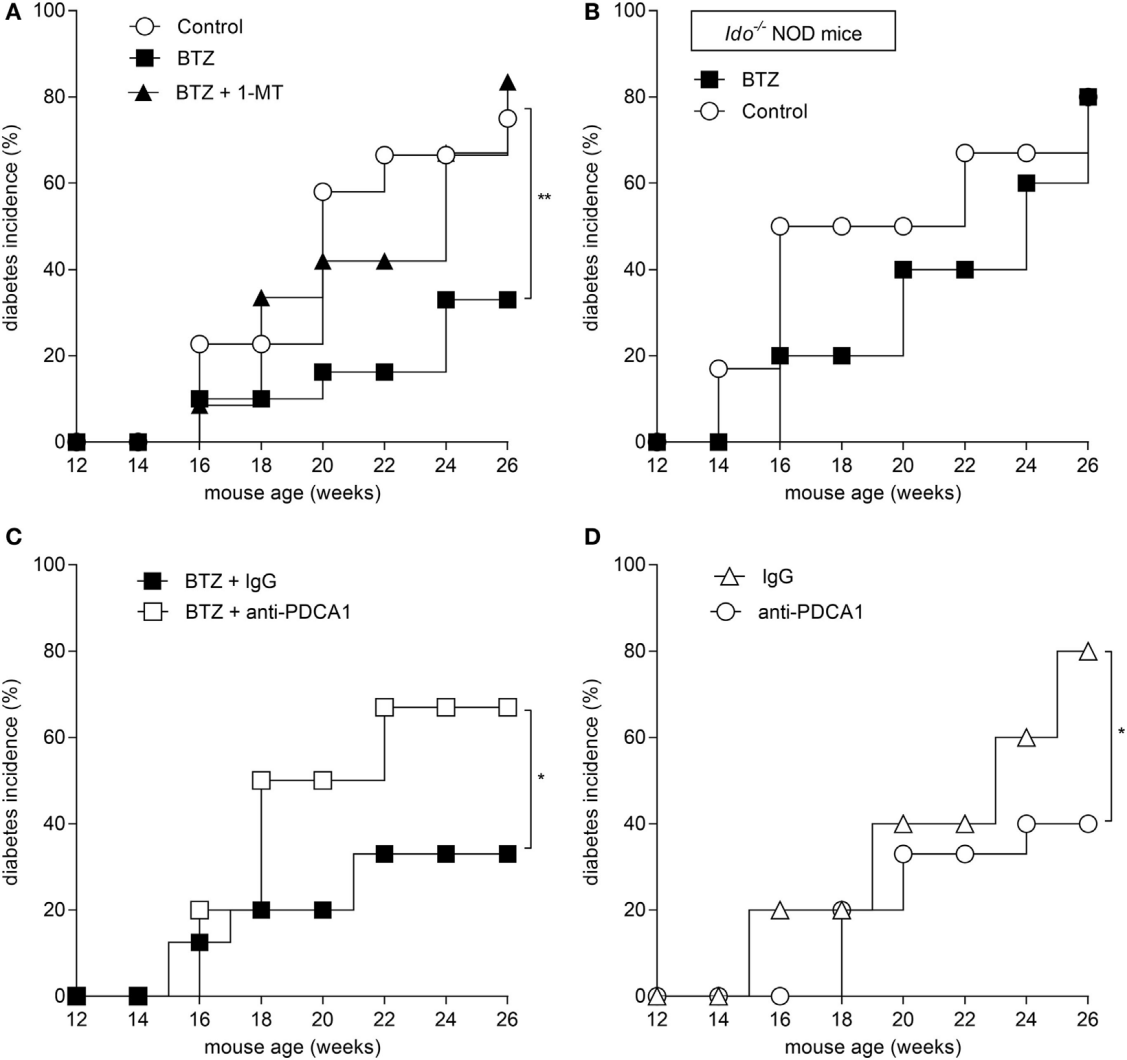
**Prevention of autoimmune diabetes by bortezomib (BTZ) requires plasmacytoid DCs and indoleamine 2,3-dioxygenase 1 (IDO1)-mediated tryptophan catabolism**. Diabetes was diagnosed in mice with blood glucose level ≥250 mg/dl and results are reported as diabetes incidence. **(A)** Prediabetic female non-obese diabetic (NOD) mice (*n* = 8) were intraperitoneally administered with vehicle (control; white circles), BTZ at 0.25 mg/kg alone (black squares) or in combination with 1-MT (black triangles), and blood glucose was monitored over time. **(B)** Prediabetic female *Ido1^−/−^* NOD mice (*n* = 8) were intraperitoneally administered with vehicle alone (control; white circles) or BTZ at 0.25 mg/kg (black squares), and blood glucose was monitored over time. **(C)** Prediabetic female NOD mice (*n* = 8) were intraperitoneally administered with BTZ at 0.25 mg/kg in combination with anti-PDCA1 depleting antibody (250 μg/mouse; white squares) or with the isotype control (rat IgG; black squares), and blood glucose was monitored over time. **(D)** Diabetic incidence of prediabetic NOD mice treated with anti-PDCA1 depleting antibody (white circles) or isotype rat IgG (white triangles). *p < 0.05 and **p < 0.01 (log-rank Mantel–Cox test).

Based on the ability of BTZ to rectify IDO1 expression and activity in pDCs *in vitro*, we evaluated whether the protective effect of the drug *in vivo* could be mediated by IDO1 and pDCs. To this purpose, mice were administered BTZ at 0.25 mg/kg as in Figure 3A, with or without 1-MT, the inhibitor of IDO1. Mice receiving vehicle alone were used as a control. IDO1 blockade completely abrogated the protective effect of BTZ (Figure 4A). Similarly, BTZ protection was lost in *Ido1^−/−^* NOD mice (Figure 4B). *In vivo* depletion of pDCs (23) occurring at the time of BTZ administration negated the protective effect of the proteasomal inhibitor against diabetes development (Figure 4C), whereas depletion of pDCs occurring before BTZ treatment would not ablate its anti-diabetic effect (data not shown). Of interest, administration of anti-PDCA1 in the absence of BTZ also significantly reduced the percentage of animals that developed diabetes over time (Figure 4D). Although difficult to explain at this time, the fact that pDC depletion *per se* exerted protective effects in untreated prediabetic NOD mice may confirm previous results indicating that pDCs can be pathogenic in NOD mice (31), and BTZ may help restoring an anti-inflammatory phenotype in NOD pDCs, so that depletion of pDCs during the treatment turns out to be detrimental as it abrogates the protective effect of BTZ.

Overall, our data indicated that proteasome inhibition is effective in the prevention of experimental autoimmune diabetes and that the effect requires pDCs and IDO1-mediated tryptophan catabolism.

### A Combination of BTZ and Suboptimal Anti-CD3 Antibody Rescues Normoglycemia in Overtly Diabetic NOD Mice

**Figure 5:**
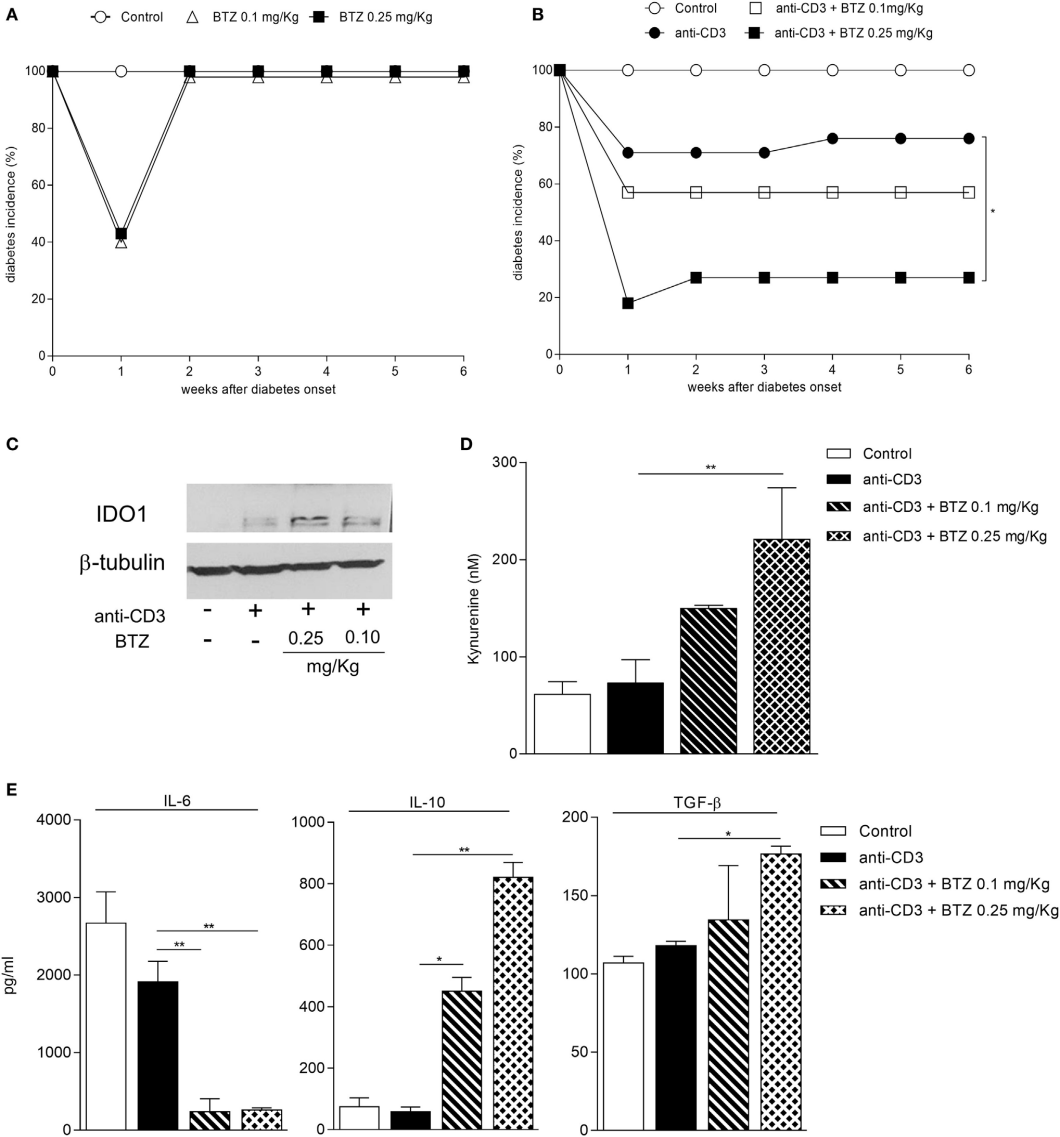
**A combination of bortezomib (BTZ) and suboptimal anti-CD3 antibody restores normoglycemia in overtly diabetic mice**. **(A)** Hyperglycemic female non-obese diabetic (NOD) mice (*n* = 8) were intraperitoneally administered with vehicle (white circles), BTZ at 0.1 mg/kg (white triangles) or 0.25 mg/kg (black squares). Blood glucose was monitored over time, and results are reported as diabetes incidence. **(B)** Hyperglycemic female NOD mice (*n* = 8) were administered with vehicle (white circles), with anti-CD3 alone (10 μg/mouse; black circles) or combined with BTZ at 0.1 mg/kg (white squares) or 0.25 mg/kg (black squares). Blood glucose was monitored over time and results are reported as diabetes incidence. *p < 0.05 (log-rank Mantel–Cox test). **(C)** Indoleamine 2,3-dioxygenase 1 (IDO1) immunoblot analysis of cell lysates from pancreatic lymph nodes (pLNs) of mice treated as in panel **(B)**. One representative experiment out of three is shown. **(D)** IDO1 activity measured in terms of kynurenine production in supernatant of cells from pLNs of mice treated as in panel **(B)** and cultured for 24 h. **p < 0.01 (one-way ANOVA and Dunnett’s multiple comparison test). **(E)** Detection of IL-6, IL-10, and TGF-β in supernatant of cells from pLNs of mice treated as in panel **(B)** and cultured for 24 h. *p < 0.05 and **p < 0.01 (one-way ANOVA and Dunnett’s multiple comparison test).

Prompted by BTZ efficacy in preventing diabetes, we interrogated whether the drug would revert an ongoing disease. NOD female mice with hyperglycemia were i.p. administered BTZ at 0.1 or 0.25 mg/kg every other day for 2 weeks. Apart from a transient and not significant reduction in glycemia at 1 week post-initiation of drug treatment, no therapeutic effect was afforded by BTZ (Figure 5A).

Previous studies have demonstrated that administration of non-mitogenic anti-CD3 represents an effective maneuver capable of rescuing normoglycemia in approximately 80% diabetic NOD mice (22). Maximum therapeutic effects are achieved at the dose of 50 µg administered i.v. for five consecutive days, and they relied on the release of TGF-β by Treg cells (32).

We thus became interested in ascertaining whether a combination of BTZ and anti-CD3 would result in additive effects. Previous experiments (Chantal Kuhn and Lucienne Chatenoud, unpublished data) found that the suboptimal dose of 10 µg is capable of reverting hyperglycemia in a minority (less than 30%) of diabetic animals (Figure 5B). In our setting, a combination of BTZ at 0.25—but not 0.1—mg/kg and 10 µg anti-CD3 restored normoglycemia in 70% diabetic mice, a value significantly different from that of mice on suboptimal anti-CD3 alone (Figure 5B).

Groups of mice, treated as in Figure 5B, were sacrificed at the end of the experiment and used as a source of pLNs to be assayed for IDO1 expression and activity as well as for cytokine production. The combination of the two drugs greatly upregulated expression of IDO1 protein (Figure 5C) and activity (Figure 5D) in pLNs, compared to suboptimal anti-CD3 alone. Perhaps most importantly, at variance with the results in Figure 3F, the combined *in vivo* treatment with BTZ and anti-CD3—but not either treatment alone—resulted in a significant increase in the production of TGF-β by pLNs, and it significantly reduced IL-6 and increased IL-10 (Figure 5E).

Because we have previously demonstrated that TGF-β is a long-term inducer of IDO1 (6), our current data would further substantiate the importance of this cytokine in the effective control of a chronic autoimmune disease such as T1D, as previously reported (33). Moreover, the inhibition of IDO1 proteasomal degradation may represent a valuable therapeutic option to be considered in associative regimens with low-dose anti-CD3, so perhaps to reduce the inflammatory side effects observed in some patients on optimal-dose anti-CD3 treatment (34).

## Discussion

Initially, the antiproliferative capacity of proteasome inhibitors has received considerable attention because of the success of their first prototypical representative, BTZ, in the treatment of B cell and plasma cell-related hematological malignancies. However, the emerging role of immunoproteasome involvement in autoantigen presentation as well as the ability of BTZ to inhibit the activation of NF-κB and suppress the release of pro-inflammatory cytokines, including IL-6, have increased interest in exploring the therapeutic potential of proteasome inhibitors in inflammatory and autoimmune disease settings (35). Selective inhibitors of the immunoproteasome and constitutive proteasome have recently been tested in models of autoimmune disease and allograft survival (17, 36). In experimental settings of inflammation and autoimmunity, BTZ does ameliorate disease severity, yet it has not been possible to identify a unitary mechanism of action, leading to the conclusion that the drug affects chronic inflammation and autoimmunity in a pleiotropic and/or model-specific fashion (37).

In the NOD mouse model system of autoimmune diabetes, we have previously demonstrated that impaired tryptophan catabolism contributes to defective tolerance to pancreatic antigens (9, 38). The condition results from a combination of functional anomalies, including poor signaling activity of IFN-γ and poor transcriptional activation of *Ido1* (4), and increased proteasomal degradation of the IDO1 protein. The latter is largely sustained by high-level production of IL-6 (8, 39), which operates through SOCS3—a negative regulator of IDO1’s enzymatic and signaling functions (40)—which acts, in turn, to ubiquitinate and degrade IDO1. One major effect of the reduced tryptophan catabolic function by IDO1 could be the defective production of kynurenine-type ligands of the aryl hydrocarbon receptor (41), which is involved in the generation of regulatory T cells (42, 43).

Plasmacytoid DCs have an ambivalent role in the pathogenesis of autoimmune diabetes (44). The protective mechanisms are thought to involve inhibition of effector T cells, induction of regulatory T cells, and production of anti-inflammatory cytokines and IDO1. Although the exact mechanism of tolerance induction by pDCs in diabetes remains to be established, the intrinsic tolerogenic properties of pDCs provide a promising, yet perhaps underestimated, target for therapeutic intervention. Here, we found that BTZ treatment *in vitro* rescued IDO1 protein expression in prediabetic NOD pDCs, which were confirmed to manifest basally high transcriptional expression of *Socs3*, β-subunits of the immunoproteasome and the pro-inflammatory pathway of NF-κB. When administered *in vivo* to NOD mice, the BTZ-conditioned pDCs blocked the immunogenic presentation of a diabetogenic peptide, and BTZ itself would oppose diabetes onset in prediabetic mice. This was reflected *in vitro* by changes in the activation patterns of NF-κB family members and in cytokine secretion profiles—most notably, IL-6—and by generation of Foxp3^+^ regulatory T cells. Nevertheless, the inhibition of IDO1 by 1-MT during the *in vitro* conditioning of pDCs with BTZ would not consistently abrogate BTZ effects, suggesting the involvement of additional mechanisms beside the IDO1-mediated effect of BTZ. Mice protected from diabetes by BTZ *in vivo* also displayed increased percentages of Foxp3^+^CD4**^+^** and IDO1 protein expression in both pLNs and pancreata, and an increased systemic Kyn/Trp ratio. The *in vivo* effect of BTZ required IDO1-mediated tryptophan catabolism and immune regulatory pDCs. Although pDC-depleted NOD mice in the absence of BTZ treatment showed a reduction in diabetes incidence, as previously reported (31), depletion of pDCs in BTZ-treated NOD mice would abrogate the protective effect of the drug. Unlike inflammatory-prone pDCs, which contribute to the pathogenesis of autoimmune diabetes, the immune regulatory properties of BTZ-conditioned pDCs are pivotal in the preventive effect of the drug in autoimmune diabetes. BTZ, however, showed no therapeutic activity when administered alone to overtly diabetic mice. Nevertheless, its combination with suboptimal dosages of autoimmune-preventive anti-CD3 antibody resulted in disease reversal in 70% diabetic mice, a therapeutic effect similar to that afforded by full-dosage anti-CD3 (22).

Clinical trials in type 1 diabetes showed that adequate dosing of anti-CD3 resulted in improved C-peptide responses and reduced exogenous insulin need (34, 45). Teplizumab, in particular, has been shown to preserve β-cell function in patients, failing, however, to represent a “cure” for patients (46). Side effects, including fever, anemia, urticarial rash, and a high incidence of anti-drug antibody development, occur frequently in patients treated with Teplizumab. For safety reasons, the dose of anti-CD3 used in the phase 3 trials was reduced as compared with that used in the pilot studies and was therefore probably insufficient (47).

Thus, effective therapies in humans that ensure efficacy while minimizing side effects remain a challenge. In addition, monotherapy with anti-CD3 also has its limits from the perspective of targeting only a single arm of the immune process. For this reason, several recent experimental studies have been exploiting synergy between anti-CD3 and various immunotherapeutic modalities (48). Our current results show that a combination of BTZ and low-dosage anti-CD3 results in a marked increase in the therapeutic efficacy of the antibody. We speculate that the synergistic effect may stem from a two-pronged approach to the mechanisms whereby NOD pDCs affect CD4^+^ T-cell differentiation toward a regulatory phenotype, by altering the cytokine milieu and the antigen presentation profile of the pDCs, *via* restoration of IDO1 functions, and by the non-lytic modulation of T cells by anti-CD3. Specifically, by reducing the proteolytic degradation of IDO1, BTZ might, at least in part, compensate for the defective *Ido1* transcription by IFN-γ as well as for the IL-6/SOCS3-driven degradation of the IDO1 protein.

In conclusion, our current data point to a potential for BTZ in the management of autoimmune diabetes in humans and, at the same time, they provide mechanistic into how the drug acts to restore immune homeostasis in autoimmune settings *via* potentiation of the IDO1 tolerogenic mechanism.

## Ethics Statement

All *in vivo* procedures were conducted in compliance with the Directive 2010/63/EU for animal experiments, according the protocols approved by the Animal Ethics Committee of the University of Perugia.

## Author Contributions

GM, EA, MP, and MB performed *in vitro* experiments; CVacca and RB performed *in vivo* experiments; CK and LC performed *in vivo* experiments with anti-CD3; CVolpi, FF, and GR performed *ex vivo* analysis; DM performed the statistical analysis; SB performed microarray analysis; LB produced *in vivo* reagents; UG and PP wrote and reviewed the manuscript; CO researched data and reviewed/edited the manuscript.

## Conflict of Interest Statement

The authors declare that the research was conducted in the absence of any commercial or financial relationships that could be construed as a potential conflict of interest.
