# Supplementary material for: The Proteasome Inhibitor Bortezomib Controls Indoleamine 2,3-Dioxygenase 1 Breakdown and Restores Immune Regulation in Autoimmune Diabetes
Source: Front Immunol. 2017 Apr 13;8:428. doi: 10.3389/fimmu.2017.00428 (PMC5390013; doi:10.3389/fimmu.2017.00428)
Supplement: Supplementary file 1 [file data_sheet_1.docx]

**Supplementary Material**

**The proteasome inhibitor bortezomib controls IDO1 breakdown and restores immune regulation in autoimmune diabetes**

*Running title*

*Proteasome inhibition in autoimmune diabetes*

Giada Mondanelli^1a^, Elisa Albini^1a^, Maria T. Pallotta^1^, Claudia Volpi^1^, Lucienne Chatenoud^2^, Chantal Kuhn^2,3^, Francesca Fallarino^1^, Davide Matino^1^, Maria L. Belladonna^1^, Roberta Bianchi^1^, Carmine Vacca^1^, Silvio Bicciato^4^, Louis Boon^5^, Giovanni Ricci^6^, Ursula Grohmann^1^, Paolo Puccetti^1^ and

Ciriana Orabona^1*^

^1^ Section of Pharmacology, Department of Experimental Medicine, University of Perugia, Perugia, Italy

^2^INSERM U1013, Hôpital Necker-Enfants Malades and Université Paris Descartes, Paris, France

^3^currently at VL37 inc., Cambridge, MA

^4^Department of Life Sciences, University of Modena and Reggio Emilia, Modena, Italy

^5^Bioceros BV, Utrecht 3584 CM, The Netherlands

^6^Animal facility of the University of Perugia, Perugia, Italy

^a^ GM end EA equally contributed to this work.

^*^Corresponding author: Ciriana Orabona, [ciriana.orabona@unipg.it](mailto:ciriana.orabona@unipg.it)

**Supplementary Informations**

**Real-Time PCR and gene expression profiling**

Real-time PCR analysis was done as described ([1-3](#_ENREF_1)) with primers specific for the immunoproteasome subunits *Psmb8/β5i*, *Psmb9/β1i, Psmb10/β2i* and *Socs3*. Results are presented as the ratio of gene expression to *Gapdh* expression, as determined by the relative quantification method (change in cycle threshold). Primers sequences are reported in Table 1.

Agilent SurePrint G3 Mouse Gene Expression 8x60K Microarrays (Agilent Technologies, Santa Clara, CA, USA) were used for microarray experiments on plasmacytoid dendritic cells (pDCs) purified from pLNs of NOD mice. Eight replicas of pDCs were prepared for each of the two conditions (early prediabetes and overtly diabetes) for a total of 16 samples. Samples were prepared according to the manufacturer’s protocols. Microarray probe fluorescence signals produced by the Agilent Feature Extraction image analysis software were converted to expression values using the R package limma (Bioconductor). Specifically, fluorescence intensities have been background corrected using the normexp method ([4](#_ENREF_4)), normalized using cyclic loess normalization, and log2 expression values for a total of 30736 probes calculated using medians of replicated non-control probes. All data analyses were performed in R version 3.2.4 using Bioconductor libraries of BioC 3.2 and R statistical packages. Microarray data are available at the following GEO accession number GSE94158. Mouse NF-kB gene set has been selected from the BioCarta NF-kB signaling pathway of The Cancer Genome Anatomy Project (https://cgap.nci.nih.gov/Pathways/BioCarta/m_nfkbPathway).

**Purification of splenic pDCs and ELISA assay**

All purification procedures involving pDCs have been previously described ([3](#_ENREF_3), [5](#_ENREF_5), [6](#_ENREF_6)). The purity of the obtained cell populations was checked by flow cytometry and was superior to 90%. Splenic pDCs, purified from prediabetic (i.e. 6 wks of age) NOD mice, were exposed for 24 hrs at 37 °C to BTZ (LC Laboratories, MA, USA) at the concentration of 10 nM in the presence or absence of l/d 1-methyl-tryptophan (1-MT; Sigma-Aldrich, St. Louis, MO), a standard IDO1 inhibitor, at the concentration of 4 µM. The supernatants of culture were harvested for cytokine detection by ELISA analysis. IL-6, IL-10 and TGF-β were analysed by specific kit (eBioscience, Inc., San Diego, CA; Promega Italia, S.r.l., Milano, Italy), according the manufacturer’s protocol.

**Table 1: Primers sequences**

| Primer | Sequence |
| --- | --- |
| *Psmb8/β5i* | F: 5’- GATGTTTTCCACTGGCAGCG -3’ |
|  | R: 5’- CACCCAACCGTCTTCCTTCA -3’ |
| *Psmb9/β1i* | F: 5’- CTGGCTGGGACCAATGTGAG -3’ |
|  | R: 5’- ATCTCGGTTCATGGCCAGAG -3’ |
| *Psmb10/β2i* | F: 5’- AACGGACCTCAGCTCTACGA -3’ |
|  | R: 5’- GCGTCATGTTTGGCTGGAAC -3’ |
| *Socs3* | F: 5’- CAGCCTGCGCCTCAAGACCTT -3’ |
|  | R: 3’- GCACCAGCTTGAGTACACAGTCG -3’ |
| *Gapdh* | F: 5’- CTGCCCAGAACATCATCCCT |
|  | R: 3’- ACTTGGCAGGTTTCTCCAGG |

**Supplementary Text**

**Gene expression profile in prediabetic versus diabetic pDCs**

We examined the expression of β1i, β2i, and β5i immunoproteasome subunits in pancreas from prediabetic female NOD mice, using conventional BALB/c as controls. Real-Time PCR assays showed significantly higher expression of the gene encoding the β5i (i.e., *Psmb8*) but not the β1i and β2i subunits of the immunoproteasome (encoded by *Psmb9* and *Psmb10*, respectively). We also found significantly higher expression of *Socs3,* the IL-6–induced molecular driver of IDO1 proteasomal degradation, in NOD pancreas ([7](#_ENREF_7)) (Supplementary Fig. 1*A*). The transcriptome analysis of pDCs purified from pLNs of early prediabetic or overtly diabetic NOD mice revealed a significantly higher expression of β1i and SOCS3 in prediabetic than diabetic pDCs. Although highly expressed, the β5i subunit was not differently expressed, and neither was subunit β2i (Supplementary Fig. 1*B*). The analysis of the gene set involved in the pro-inflammatory signaling pathway of NF-kB revealed a higher expression of the pathway in prediabetic than diabetic pDCs (Supplementary Fig. 1*C*). Overall, the transcriptome analysis in the pancreas and pancreatic pDCs suggested a role for proteasomal degradation of IDO1 in the early phase of the disease, when pDCs express a more inflammatory-prone phenotype.

***In vitro* cytokines production by prediabetic NOD pDCs.**

Splenic pDCs isolated from prediabetic NOD mice (i.e. 6 wks of age) were *in vitro* treated with BTZ alone or in combination with 1-MT, the standard inhibitor of IDO1 enzyme, and assayed for the release of IL-6, IL‑10 and TGF-β. At 24 h, BTZ caused a significant decrease in the production of pro-inflammatory IL-6, as well as an increase in anti-inflammatory IL‑10, no effect of BTZ exposure was instead observed on the anti-inflammatory cytokine TGF-β (Supplementary Fig. 2). Interestingly, the inhibition of IDO1 enzyme by 1-MT during the *in vitro* conditioning of pDCs by BTZ, selectively negated the drug effect on IL-10 secretion (Supplementary Fig. 2), suggesting that intact IDO1 up-regulation by BTZ could play a role in controlling the immunoregulatory cytokine in prediabetic NOD pDCs.

**Supplementary Figures with Legends**


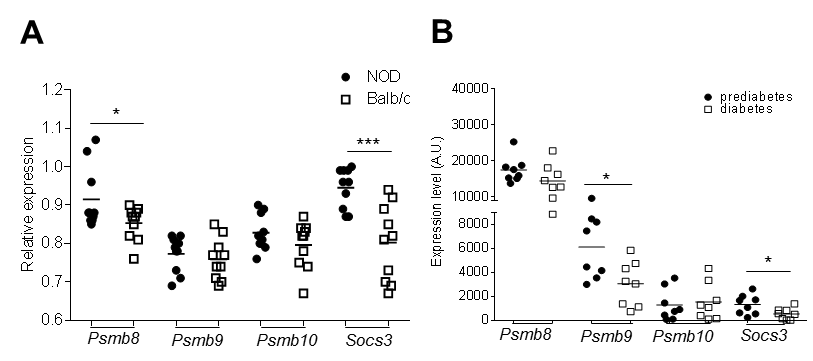

**C**

**Supplementary Figure 1**: Gene expression of the immunoproteasome subunits and *Socs3*. (A) Real-Time PCR analysis of *Psmb8*, *Psmb9*, *Psmb10* (encoding β5i, β1i and β2i immunoproteasome subunits, respectively) and *Socs3* transcripts in pancreas from female NOD mice (n=10; black circles), normalized to the expression of *Gapdh* (encoding glyceraldehyde phosphate dehydrogenase) and using conventional BALB/c as controls (n=10; white squares). Results are presented as the ratio of gene expression to *Gapdh* expression, as determined by the relative quantification method (change in cycle threshold). *p ˂ 0.05; *** p ˂0.001 (NOD *versus* control mice). (B) Transcriptome analysis of pDCs purified from pancreatic lymph nodes of prediabetic (n=8; black circles) and diabetic (n=8; white squares) NOD mice. (C) Average gene-expression values of NF-kB signaling pathway in prediabetic (n=8) and diabetic (n=8) pDCs purified from NOD pancreatic lymph nodes. Data are shown as mean ± standard error of the mean (SEM).

**
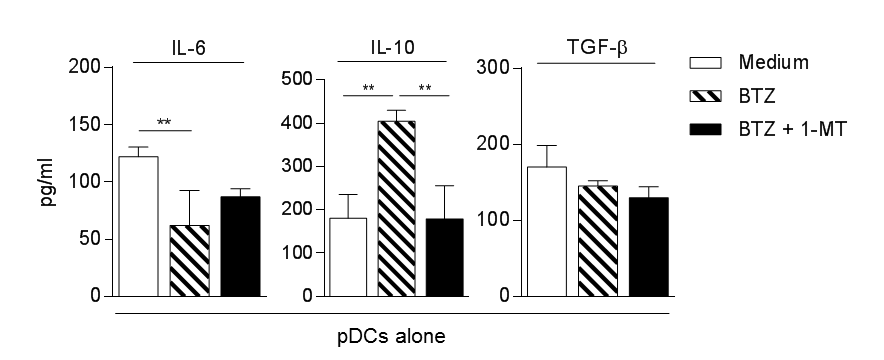
**

**Supplementary Figure 2:** Cytokines production by prediabetic NOD pDCs. Secretion of IL-6, IL‑10 and TGF-β in supernatants of pDCs left untreated (medium) or treated with BTZ alone or in combination with 1-MT for 24h. ** p ˂ 0.01 (one-way ANOVA followed by Bonferroni multiple comparison test).

**References**

1. Albini E, Rosini V, Gargaro M, Mondanelli G, Belladonna ML, Pallotta MT, et al. Distinct roles of immunoreceptor tyrosine-based motifs in immunosuppressive indoleamine 2,3-dioxygenase 1. *Journal of cellular and molecular medicine* (2017) **21**(1):165-76. Epub 2016/10/04. doi: 10.1111/jcmm.12954. PubMed PMID: 27696702; PubMed Central PMCID: PMCPmc5192792.

2. Belladonna ML, Volpi C, Bianchi R, Vacca C, Orabona C, Pallotta MT, et al. Cutting edge: Autocrine TGF-beta sustains default tolerogenesis by IDO-competent dendritic cells. *Journal of immunology* (2008) **181**(8):5194-8. PubMed PMID: 18832670.

3. Pallotta MT, Orabona C, Volpi C, Vacca C, Belladonna ML, Bianchi R, et al. Indoleamine 2,3-dioxygenase is a signaling protein in long-term tolerance by dendritic cells. *Nature immunology* (2011) **12**(9):870-8. doi: 10.1038/ni.2077. PubMed PMID: 21804557.

4. Silver JD, Ritchie ME, Smyth GK. Microarray background correction: maximum likelihood estimation for the normal-exponential convolution. *Biostatistics (Oxford, England)* (2009) **10**(2):352-63. Epub 2008/12/11. doi: 10.1093/biostatistics/kxn042. PubMed PMID: 19068485; PubMed Central PMCID: PMCPmc2648902.

5. Pallotta MT, Orabona C, Bianchi R, Vacca C, Fallarino F, Belladonna ML, et al. Forced IDO1 expression in dendritic cells restores immunoregulatory signalling in autoimmune diabetes. *Journal of cellular and molecular medicine* (2014) **18**(10):2082-91. doi: 10.1111/jcmm.12360. PubMed PMID: 25215657; PubMed Central PMCID: PMC4193887.

6. Grohmann U, Fallarino F, Bianchi R, Orabona C, Vacca C, Fioretti MC, et al. A defect in tryptophan catabolism impairs tolerance in nonobese diabetic mice. *The Journal of experimental medicine* (2003) **198**(1):153-60. doi: 10.1084/jem.20030633. PubMed PMID: 12835483; PubMed Central PMCID: PMC2196078.

7. Orabona C, Pallotta MT, Volpi C, Fallarino F, Vacca C, Bianchi R, et al. SOCS3 drives proteasomal degradation of indoleamine 2,3-dioxygenase (IDO) and antagonizes IDO-dependent tolerogenesis. *Proceedings of the National Academy of Sciences of the United States of America* (2008) **105**(52):20828-33. doi: 10.1073/pnas.0810278105. PubMed PMID: 19088199; PubMed Central PMCID: PMC2634889.
